# Clinical Lectures on Diseases of the Eye

**Published:** 1864-12

**Authors:** E. L. Holmes

**Affiliations:** Lecturer on Diseases of the Eye and Ear in Rush Medical College, and Surgeon of the Chicago Charitable Eye and Ear Infirmary


### CLINICAL LECTURES ON DISEASES OF THE EYE.

By E. L HOLMES, M. D., of Chicago,

*Lecturer on Diseases of the Eye and Ear in Rush Medical College, and Surgeon of the Chicago Charitable Eye and Ear Infirmary.*

### ARTIFICIAL PUPIL.

*Gentlemen:*—Whenever, under certain circumstances, the natural opening in the iris becomes obstructed, thereby shutting the rays of light from the retina, a new or artificial pupil may be made in the iris and the usefulness of the eye restored.

The operation by which this is usually done is termed Iridectomy, and is often followed by the most satisfactory results. This subject opens a long chapter in Ophthalmic surgery, of which I can only give you the leading principles. The operation is especially applicable to three classes of cases. 1st. Those in which light is prevented from entering the eye, by opacities of the cornea, central opacities of the lens, and closure of the pupil by organized lymph. 2d. Those in which experience has shown, without, however, giving perfectly satisfactory reasons, that this operation arrests the progress of a destructive inflammation of the iris, choroid, and ciliary processes, relieving pain and, in some instances, restoring sight. 3d. Certain cases of cataract in which, in the operation by extraction, the passage of the lens through the iris (pupil) may be facilitated by enlarging the natural pupil, thereby reducing the danger of injury to the iris.

The operation is performed in certain other conditions of the eye ; but a careful study of the three classes just mentioned will enable you to appreciate the indications for the operation in other cases.

Before performing the operation for artificial pupil in the first and third classes of cases, it is a matter of importance to examine the condition of the retina. You can readily understand, that, whenever in these cases, the retina or optic nerve is amaurotic, there would probably be little advantage derived from the operation.

Whenever there is an opacity of the cornea front of the pupil, and the patient can distinguish objects held in the plain of the iris, there is almost a certainty that the retina is intact. If there are adhesions between the iris and cornea or lens, with opacities of either of these organs, there is reason to believe the retina is healthy, if the iris retains its normal color, if the patient can distinguish the light of a candle in the dark, at the distance of thirty feet, or if he can perceive the shadow of the hand passed to and fro before the eye.

In cases of extensive adhesions between the iris and lens, especially where the iris is discolored, or the eye atrophied, there is reason to fear that both the choroid and retina are impaired. Still even in very unpromising cases of this kind, the removal of a piece of the iris has sometimes restored quite good vision to the eye.

As I have already informed you, chronic iritis, with adhesions to the lens, is very liable to suffer relapses, terminating finally in choroiditis. The removal of a part of the iris often arrests this tendency.

In Glaucoma, and certain forms of choroiditis, Iridectomy is one of two operations, which are most certain of affording relief. For explanations of the curative effects of this operation in chronic iritis and glaucoma, I would refer you to several articles by Graefe, a translation of which may be found in the publications of the Sydenham Society. In the last few numbers of Braithwaite's Retrospect is quite a full discussion of the subject.

It is sometimes a question of considerable importance to decide whether an operation on one eye will prove advantageous when the other is perfect. This question, however, can scarcely arise when it is the object of the operation to arrest inflammation.

In other cases as, for instance, in opacities of the cornea, good authorities differ in opinion as to the advantage to be derived from an operation on one eye, the other being sound. Inasmuch as the new pupil is not situated in the natural axis of vision, there is sometimes difficulty on the part of patients in directing the eyes upon an object without double vision, when one eye is sound and the other has an artificial pupil. Although patients after a time learn to overcome this disadvantage, I prefer not to operate where one eye is normal, unless the new pupil can be placed quite near the centre of the iris, and a large part of the cornea is perfectly transparent. Where both eyes are quite diseased but more or less suitable for the operation, I believe it is usually preferable to operate upon the better one alone.

A question sometimes arises in reference to the most suitable position for the new pupil. In many cases of opacity of the cornea there is but one choice, since but one portion of the cornea, and that often a minute portion, may be left transparent. Of course the section of the iris behind this transparent portion of the cornea can alone be removed with advantage. Whenever, as in central opacities of the cornea or lens, an artificial pupil can be formed in any section of the iris, there are reasons why the opening should be made in its inner, or inner and lower quarter. Some prefer to excise the upper, and others the lower part of the iris. Except when absolutely necessary, the new pupil should not be placed towards the external angle of the eye. In all cases it should be placed as near the centre of the cornea (iris) as possible.

Reference to the three cases of artificial pupil which I have been able to bring before you during the winter, will serve to impress upon your memory some of the foregoing principles. In the case of the young man, who lost his sight by the premature discharge of a blast, so much of the cornea was cicatrized that the artificial pupil could only be made in the upper and inner sixth of the right iris.

The German, who lost the use of his eyes from muco-p uruulcent conjunctivitis, is able to see quite well through a pupil in the upper part of the iris, made behind a very narrow rim of transparent cornea.

The Irishman, with the very dense albugo, covering more than three-fourths of the right cornea, has obtained good vision by means of a pupil in the outer quarter of the iris. I believe you will seldom find a patient with an artificial pupil, *in the outer quarter of the iris*, who sees better than the one last mentioned.

It is well to remember that, where a small transparent portion of the cornea is surrounded by a translucent rim, vision is liable to be quite indistinct, after an operation for artificial pupil, on account of the diffraction of light. In these cases a minute aperture in a thin plate of dark metal, held close before the cornea, will generally improve vision by preventing diffusion of light.

The operation itself is usually simple, and attended with very little difficulty. With the use of chloroform and the ordinary instruments to keep the lids open, the eye can be fixed in any desired position, with a pair of forceps holding a small fold of conjunctiva. By means of a lance-shaped knife an incision, one to two lines long, is made into the anterior chamber, by passing the point of the knife, held with its flat surface parallel with the plane of the iris, through the sclerotic, a little less than a line behind its union with the cornea. After withdrawing the knife *slowly* to prevent a rapid discharge of the aqueous humor, and consequent sudden reduction of the intraocular pressure upon the delicate vessels of the retina, and to avoid sudden movement of the lens and iris forward, the fine iris forceps is introduced into the anterior chamber, without pressure upon the iris and lens; the forceps should be opened so as to allow a fold of the iris, near its papillary edge, to enter between the blades; they should then be closed and withdrawn with a piece of the iris, without tearing it from its ciliary attachments; this piece should be clipped off with a pair of scissors close to the cornea.

The anterior chamber is often filled with blood after this operation, but it is usually all absorbed in less than forty-eight hours. The only dressing necessary is a bandage with compresses to keep up gentle pressure upon the eye for a day or two. Iridectomy is rarely followed by inflammation either of the iris or other tissues.

It is an important fact for you to bear in mind that tearing or cutting out a piece of iris is scarcely ever followed by inflammation, but punctures and bruises of the iris, and contact of pieces of cataract and foreign substances with the iris, are almost sure to lead to serious inflammation.

In cases of central opacities of the cornea and lens, the operation of Iridodesis has been recommended in place of Iridectomy. This operation consists in seizing a small fold of the iris and drawing it through the wound in the cornea without tearing the fibres of the iris. Instead of snipping off the piece of iris, a fine thread is tied around it and left to slough off, when the iris becomes fixed in the cicatrix. In this way the normal pupil is preserved, but drawn from the opacity of the iris or lens and left where the cornea or lens is transparent. It is stated on good authority that while this operation is more difficult, it is also much more liable to cause inflammation than Iridectomy.

A few important points regarding Iridectomy in Glaucoma will be mentioned at another time.

